# 3D Visualization of Dynamic Cellular Reaction of Pulpal CD11c+ Dendritic Cells against Pulpitis in Whole Murine Tooth

**DOI:** 10.3390/ijms222312683

**Published:** 2021-11-24

**Authors:** Sujung Hong, Yeoung-Hyun Park, Jingu Lee, Jieun Moon, Eunji Kong, Jehwi Jeon, Joo-Cheol Park, Hyung-Ryong Kim, Pilhan Kim

**Affiliations:** 1 Graduate School of Nanoscience and Technology, Korea Advanced Institute of Science and Technology (KAIST), 291 Daehak-ro, Yuseong-gu, Daejeon 34141, Korea; sujungh@kaist.ac.kr (S.H.); whiteguy6561@kaist.ac.kr (J.L.); jieunhye@kaist.ac.kr (J.M.); 2 KI for Health Science and Technology (KIHST), Korea Advanced Institute of Science and Technology (KAIST), 291 Deahak-ro, Yuseong-gu, Daejeon 34141, Korea; kong5202@kaist.ac.kr (E.K.); theaitetos@kaist.ac.kr (J.J.); 3 Department of Oral Histology-Developmental Biology, School of Dentistry and Dental Research Institute, Seoul National University, Seoul 03080, Korea; pyh5436@snu.ac.kr (Y.-H.P.); jcapark@snu.ac.kr (J.-C.P.); 4 Graduate School of Medical Science and Engineering, Korea Advanced Institute of Science and Technology (KAIST), 291 Daehak-ro, Yuseong-gu, Daejeon 34141, Korea; 5 Department of Pharmacology, College of Dentistry, Jeonbuk National University, Jeonju 54896, Korea

**Keywords:** imaging, immunity, inflammation, pulpitis, dentin

## Abstract

In dental pulp, diverse types of cells mediate the dental pulp immunity in a highly complex and dynamic manner. Yet, 3D spatiotemporal changes of various pulpal immune cells dynamically reacting against foreign pathogens during immune response have not been well characterized. It is partly due to the technical difficulty in detailed 3D comprehensive cellular-level observation of dental pulp in whole intact tooth beyond the conventional histological analysis using thin tooth slices. In this work, we validated the optical clearing technique based on modified Murray’s clear as a valuable tool for a comprehensive cellular-level analysis of dental pulp. Utilizing the optical clearing, we successfully achieved a 3D visualization of CD11c+ dendritic cells in the dentin-pulp complex of a whole intact murine tooth. Notably, a small population of unique CD11c+ dendritic cells extending long cytoplasmic processes into the dentinal tubule while located at the dentin-pulp interface like odontoblasts were clearly visualized. 3D visualization of whole murine tooth enabled a reliable observation of these rarely existing cells with a total number less than a couple of tens in one tooth. These CD11c+ dendritic cells with processes in the dentinal tubule were significantly increased in the dental pulpitis model induced by mechanical and chemical irritation. Additionally, the 3D visualization revealed a distinct spatial 3D arrangement of pulpal CD11c+ cells in the pulp into a front-line barrier-like formation in the pulp within 12 h after the irritation. Collectively, these observations demonstrated the unique capability of optical clearing-based comprehensive 3D cellular-level visualization of the whole tooth as an efficient method to analyze 3D spatiotemporal changes of various pulpal cells in normal and pathological conditions.

## 1. Introduction

Teeth are under continuous challenges by foreign pathogens and metabolic products of oral bacteria [1,2,3,4,5]. The odontoblasts sense infiltrating pathogens and secrete proinflammatory cytokines which trigger the initial innate inflammatory response with the pulpal dendritic cells [6,7,8]. Dendritic cells can be recruited and uptake foreign antigens by phagocytosis [9,10]. In experimental dental caries or pulpitis-induced teeth, pulpal dendritic cells were observed to be accumulated along the pulp-dentin border [11,12,13]. Zhang et al. (2006) reported that CD11c+ dendritic cells exist in the dental pulp and work as sentinel cells quickly migrating to the pulp-dentin interface near the irritated side of the teeth [14]. Furthermore, Bhingare et al. (2014) reported that the dental pulp dendritic cells can migrate to regional lymph nodes for antigen presentation to T cells with increased expression of MHC class II [15]. Collectively, these studies demonstrate that the highly dynamic cooperation of odontoblasts and pulpal dendritic cells orchestrate the initial innate immune responses and the subsequent adaptive immune response [5,16,17]. Additionally, using pulpitis-induced rodent models, many studies have been actively conducted to dissect the cellular and molecular mechanism underlying dental pulp immunity. However, most analyses have been performed by conventional immunohistochemical analysis [14,15,18,19] or FACS analysis [16,20,21] which have provided limited information about the cellular-level 3D spatial distribution of various pulpal immune cells in the dentin-pulp complex of the whole tooth. Recently, we established an optimized tooth optical clearing method based on modified Murray’s clear and showed a 3D cellular-level visualization of the whole tooth by using various transgenic reporter mice expressing endogenous fluorescence protein in a specific subpopulation of immune cells [22,23].

In this work, a 3D cellular-level visualization of CD11c+ cells in the dentin-pulp complex was achieved by optically clearing the whole intact tooth of CD11c-YFP transgenic mouse [24]. Notably, the 3D visualization of the whole murine tooth clearly revealed a small population of CD11c+ cells with a total number less than a couple of tens in one tooth. They have an odontoblast-like morphology extending long cytoplasmic processes into the dentin identified by a second harmonic generation signal from the dentinal collagen. They were located at the dentin-pulp interface like odontoblasts but did not express odontoblast differentiation markers, dentin matrix protein 1 (DMP1), and Nestin. In the dental pulpitis model induced by burring and acid etching, the CD11c+ dendritic cells at the dentin-pulp interface were significantly increased. Additionally, the 3D cellular-level visualization of the whole irritated tooth clearly showed that the pulpal CD11c+ cells were accumulated at the irritated pulp site showing acute blood vessel impairment within 12 h. The CD11c+ cells were spatially arranged in 3D pulp space with a distinct front-line barrier-like formation, suggesting their active role in the immune defense at an early stage.

## 2. Results

### 2.1. 3D Cellular-Level Visualization and Identification of CD11c+ Cells in the Dentin-Pulp Complex

**Figure 1:**
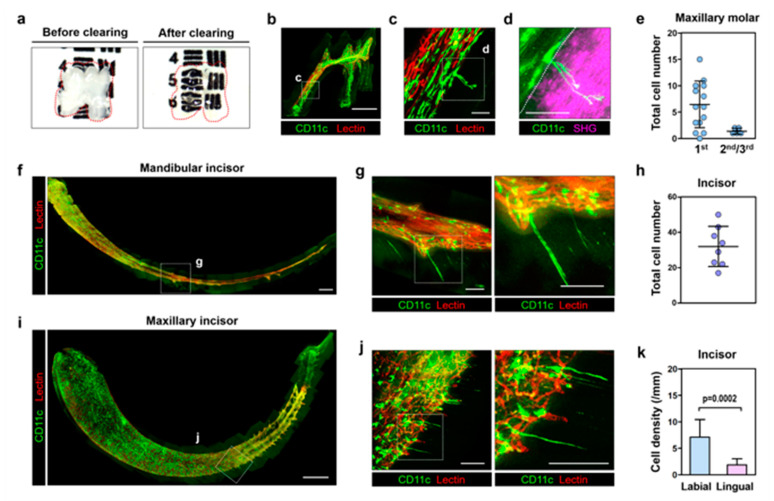
Identification of CD11c+ cells extending cytoplasmic processes into dentinal tubules. (**a**) Photograph 1st of a mandibular molar before and after optical clearing. (**b**) Maximal intensity projection images of 1st maxillary molar extracted from CD11c-YFP mouse showing CD11c-YFP cells (green) and vessels labeled by intravenously injected tomato lectin (red), Scale bar, 500 μm. (**c**) Magnified image from dotted box in (**b**). Scale bar, 50 μm. (**d**) Maximal intensity projection image with SHG signal (magenta) of dentinal collagen from dotted box in (**c**). Scale bar, 50 μm. (**e**) Total cell number of CD11c+ cells at the dentin-pulp interface in maxillary molars (1st maxillary molar; *n* = 15, 2nd/3rd maxillary molar; *n* = 6). (**f**) Maximal intensity projection image of mandibular incisor from CD11c-YFP mouse. Scale bar, 500 μm. (**g**) Magnified images from dotted box in (**f**). Scale bar, 100 μm. (**h**) Total cell number of CD11c+ cells at the dentin-pulp interface in incisors (*n* = 8). (**i**) Maximal intensity projection image of maxillary incisor from CD11c-YFP mouse. Scale bar, 500 μm. (**j**) Magnified images from dotted box in (**i**). Scale bar, 100 μm. (**k**) Cell density of CD11c+ cells at the dentin-pulp interface in the labial part and lingual part of incisors (*n* = 10). Statistical analysis was conducted with paired two-tailed *t*-tests.

**Figure 2:**
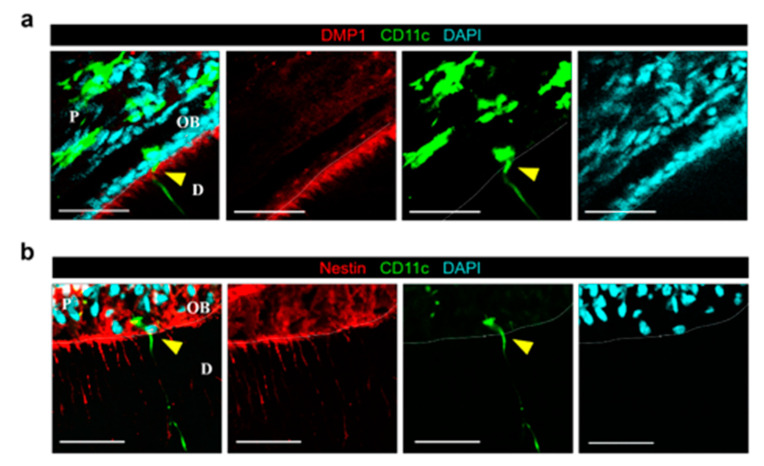
CD11c+ cell at dentin-pulp interface does not express odontoblast differentiation markers of DMP1 and Nestin. (**a**) Single-plane images of the tooth extracted from CD11c-YFP mouse after immunostaining of DMP1. Yellow arrowhead marks CD11c+ cell at the dentin-pulp interface. Scale bar, 50 μm. DMP1 (red), CD11c (green), DAPI (cyan). (**b**) Single plane images of the tooth extracted from CD11c-YFP mouse after immunostaining of Nestin. Yellow arrowhead marks CD11c+ cell at the dentin-pulp interface. Scale bar, 50 μm. Nestin (red), CD11c (green), DAPI (cyan). White dotted lines delineate the dentin-pulp interface. *D*: dentin, *P*: pulp, *OB*: odontoblast layer.

After the optical clearing procedure [22], the 1st molar extracted from the CD11c-YFP mouse became highly transparent (Figure 1a), enabling a 3D multi-color cellular-level visualization of the whole intact molar with optical sectioning confocal and two-photon laser-scanning microscope (Figure 1b–d, Supplementary Video S1). A similar optical clearing procedure successfully clarified both the mandibular and maxillary incisors (Supplementary Figure S1). Pulpal CD11c+ cells with various shapes and blood vessels were clearly visualized at the cellular level with YFP and tomato lectin, respectively (Figure 1c). Notably, a small population of CD11c+ cells were observed with an odontoblast-like morphology extending long cytoplasmic processes into the dentinal tubule (Figure 1c,d and Supplementary Video S1). The dentin was identified by dentinal collagen visualized with a second harmonic generation (SHG) signal [25,26] (Figure 1d, Supplementary Video S2), and the CD11c+ cells extending long processes into the dentinal tubule were located at the dentin-pulp interface. The average number of CD11c+ cells at the dentin-pulp interface observed was relatively small, 6.47 ± 4.41, in the 1st maxillary molars (*n* = 15) and only 1.33 ± 0.52 in the 2nd/3rd maxillary molars (*n* = 6) shown in Figure 1e. On the other hand, in the incisor, the CD11c+ cells at the dentin-pulp interface were more abundantly observed in both of the mandibular and maxillary incisors (Figure 1f–j) with an average number of 29.9 ± 10.99 (*n* = 8) shown in Figure 1h. Additionally, more CD11c+ cells along the dentin-pulp interface were observed in the labial parts (7.09 ± 3.33/mm, *n* = 10) than in the lingual parts of the incisors (1.86 ± 1.16/mm, *n* = 10) shown in Figure 1k. In contrast to odontoblasts which have a single cellular process, some of the CD11c+ cells at the dentin-pulp interface had two to three cytoplasmic processes extending into a dentinal tubule. (Figure 1d,f). Although located at the dentin-pulp interface like odontoblasts, the CD11c+ cells did not express odontoblast differentiation markers including DMP1 and Nestin [27,28] shown by conventional immunofluorescence staining of teeth sections from the CD11c-YFP mice (Figure 2). 

### 2.2. 3D Cellular-Level Visualization of the Spatial Arrangement of Pulpal CD11c+ Cells against Acute Pulpitis

**Figure 3:**
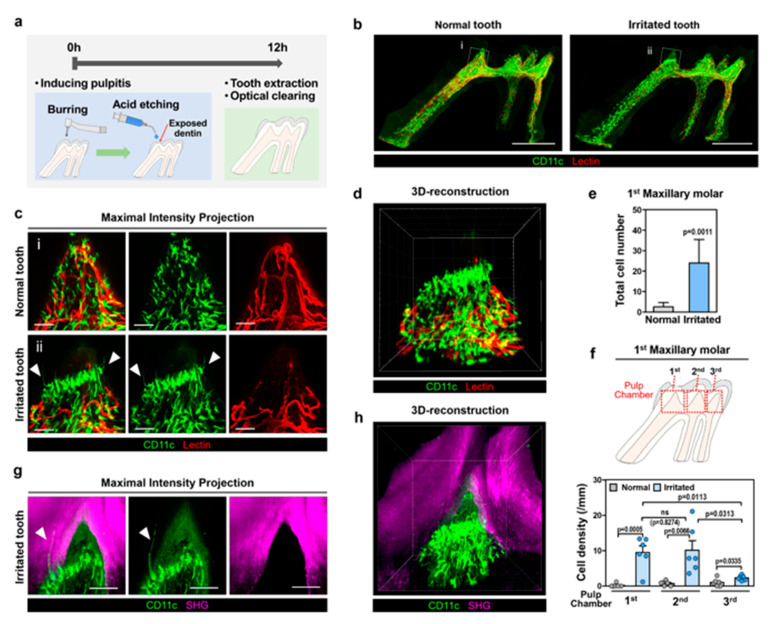
3D cellular-level visualization and quantification of CD11c+ cells after acute pulpitis induction. (**a**) Schematic illustration of acute pulpitis induction. (**b**) Maximal intensity projection images of normal and irritated tooth extracted from CD11c-YFP transgenic mouse showing CD11c-YFP cells (green) and vessels labeled by intravenously injected tomato lectin (red). Scale bar, 500 μm. (**c**) Magnified images from dotted box in (**b**). Arrowheads mark CD11c+ cells at dentin-pulp interface. Scale bar, 50 μm. (**d**) 3D reconstructed image of the irritated tooth in (**c**) (Supplementary Video S2). (**e**) Total cell number of CD11c+ cells at dentin-pulp interface in the crown of normal and irritated teeth (*n* = 6, each). (**f**) Cell density of CD11c+ cells at dentin-pulp interface in 1st, 2nd, 3rd pulp chamber compartment in normal and irritated teeth (*n* = 6, each). Statistical analysis for the comparison between normal and irritated tooth was conducted with unpaired two-tailed *t*-tests. Statistical analysis for the comparison between pulp chamber compartment in irritated teeth was conducted with paired two-tailed *t*-test. (**g**) Maximal intensity projection images of the irritated tooth in (**c**) with SHG signal (magenta) of dentinal collagen. Arrowhead marks CD11c+ cell at dentin-pulp interface. Scale bar, 50 μm. (**h**) 3D reconstructed image of (**g**) (Supplementary Video S5).

The irritated tooth with acute pulpitis was successfully visualized at the cellular level after optical clearing (Figure 3a,b). Magnified images of the pulp horn under the burred cusp of the irritated tooth (marked by a dotted box in Figure 3b) showed a greatly increased number of CD11c+ cells at the dentin-pulp interface extending cytoplasmic processes into the dentinal tubule (Figure 3c, Supplementary Video S3). Notably, a distinct spatial arrangement of the pulpal CD11c+ cells in a barrier-like formation was observed at the irritated pulp site with impaired vessels visualized by intravenously injected tomato lectin (Figure 3d, Supplementary Video S4). Concurrently, the total number of CD11c+ cells at the dentin-pulp interface in the crown pulp also was significantly increased in the irritated molar (24 ± 11.45, *n* = 6) compared to the normal molar (2.5 ± 2.17, *n* = 6) (Figure 3e). We further divided the pulp chamber into 3 compartments and quantified the density of the CD11c+ cells along with the dentin-pulp interface (Figure 3f). In the irritated tooth in comparison to the untreated normal tooth, the CD11c+ cells at the dentin-pulp interface were greatly increased in all 3 compartments while the increment was significantly bigger in the 1st and 2nd compartments near the burred cusp. In addition, the pulp horn under the burred cusp of the irritated tooth was imaged by a two-photon microscope (Figure 3g,h and Supplementary Video S5). The maintenance of the dentin was clearly visualized by the SHG signal of the dentinal collagen. The CD11c+ cells with extended cytoplasmic processes into the dentin were observed.

## 3. Discussion

In this work, we successfully achieved a 3D cellular-level visualization of a whole intact tooth extracted from a CD11c-YFP mouse expressing YFP mostly in mononuclear phagocytes [24]. The entire dentin-pulp complex could be thoroughly observed in cellular resolution. Notably, we could identify a unique small population of CD11c+ cells with a total number less than a couple of tens in one tooth. These CD11c+ cells were specifically located along the dentin-pulp interface and had a distinct morphology like odontoblasts which extended very long cytoplasmic processes into the dentinal tubule (Figure 1 and Supplementary Video S1). Similar to odontoblasts, CD11c+ cells are located at the dentin-pulp interface but do not express the odontoblast differentiation markers, DMP1 and Nestin [27,28], suggesting they are a different type of cell (Figure 2). Furthermore, some of them have multiple long processes (Figure 1d,f) in contrast to conventional odontoblasts with single cellular process protruding into the dentinal tubule [29,30]. Notably, we also observed similar odontoblast-like shaped cells in the optically cleared tooth extracted from the CX3CR1-GFP mouse and CSF1R-GFP mouse (Supplementary Figure S2). In these mice, GFP is expressed in the subsets of the myeloid lineage immune cells including mononuclear phagocytes, dendritic cells, macrophages, and monocytes [31,32,33,34]. Nevertheless, the strong expression of CD11c-YFP, commonly expressed in a subpopulation of immune cells including monocytes, macrophages, and dendritic cells, suggests their potential role in the immune response. Indeed, in the acute pulpitis model, the number and density of CD11c+ cells at the dentin-pulp interface greatly increased (Figure 3c,e and Supplementary Video S3), particularly at the adjacent pulp chamber compartments (Figure 3f). In addition, the 3D cellular-level visualization clearly revealed a distinct spatial arrangement of the pulpal CD11c+ cells in a barrier-like formation at the irritated pulp sites within 12 h after inducing acute pulpitis (Figure 3d and Supplementary Video S4), suggesting their potential role in immune responses. At the pulp area above the barrier-like arrangement of pulpal CD11c+ cells, no blood vessels labeled by intravenously injected tomato lectin were observed. This might be due to a localized vascular collapse with increases in interstitial pressure in the dental pulp. Notably, the increased extracellular fluid pressure is associated with the inflammatory response against penetrating pathogens as a mechanism of host defense [35]. In contrast, a normal vasculature labeled with intravenously injected tomato lectin was observed below the barrier-like arrangement of pulpal CD11c+ cells, suggesting that the barrier-like formation of the distinct pulpal CD11c+ cells may act as a protector for the pulpal cells against excessive tissue damage due to the inflammatory response. Notably, recent reports showed that CD11c+ cells are critically involved in the early activation of the wound healing process using a CD11c-DTR mouse for the depletion of CD11c+ cells [36]. 

Nevertheless, the cellular identity of these greatly increased pulpal CD11c+ cells with an arrangement in a barrier-like formation (Figure 3d and Supplementary Video S3) remains undetermined and requires further investigation. One candidate for their identity is macrophages known to have a pivotal role in dentin repair in a pulpitis murine model by mediating dental pulp stem cell activity [37]. Macrophages, one of the mononuclear phagocytes, are immune cells essential for both the inflammatory response (M1-like) and anti-inflammatory response (M2-like) [38]. During the inflammatory response, macrophages are actively recruited for the phagocytosis of dead cells and debris and then can be converted into anti-inflammatory or suppressive cells in response to local microenvironmental signals and promote tissue repair for wound healing [39]. Another candidate is dendritic cells known to be the first responding cells migrating to the odontoblast layer when dental caries occur, and the odontoblast is destroyed by various factors such as bacteria toxin [9,10,40]. CD11c+ dendritic cells in the dental pulp migrate to the site closest to the external stimulus, sense and uptake the microbial invasion, and act as sentinel cells in the first line of defense [14]. A portion of the greatly increased pulpal CD11c+ cells arranged in the barrier-like formation in the pulp of the acute pulpitis model might be these previously identified sentinel CD11c+ dendritic cells. The significantly increased CD11c+ cells at the dentin-pulp border extending long cytoplasmic processes into the dentinal tubule may be the sentinel CD11c+ dendritic cells in the action of capturing pathogens. These CD11c+ dendritic cells can migrate to the draining lymph nodes for antigen processing and presentation at the later stage of the immune response [14]. Reportedly, in rat and human teeth, the expression of putative dendritic cell markers was observed in several cells extending cytoplasmic processes into dentinal tubules at the dentin-pulp interface [19,41,42]. These cells also increased at the early time points in the pathological condition, suggesting their involvement in the initial defense role in the pulpal immune response.

Despite continuous efforts, the cellular and molecular mechanism underlying the immune response in the dentin-pulp complex remains not fully understood. It is partly due to the technical difficulty in the detailed 3D cellular-level observation of a whole intact tooth. Indeed, conventional histological analysis of thin tooth sections has limitations in offering a comprehensive 3-dimensional view of the cellular structure of dental pulp and systematic observation of the spatial distribution of various pulpal cells dynamically changing in pathological conditions [14,15,18,19]. As demonstrated in this work, the 3D cellular-level visualization based on optical clearing technique can enable a thorough observation of the entire dentin-pulp complex of the whole tooth in cellular resolution. Yet, several limitations such as technical difficulty in fluorescent labeling specific types of cells in the entire dentin-pulp complex and potential loss of fluorescence signal of fluorophore during optical clearing procedure are remained and need further development. In this study, we mostly utilized genetically engineered mice endogenously expressing reporter fluorescence proteins, YFP or GFP, in specific cells such as CD11c+ cells, CX3CR1+ cells, and CSF1R+ cells, thereby bypassing the technical difficulty in fluorescence labeling of the whole intact tooth with an exogenous fluorophore. Additionally, the blood vessel endothelial cell was relatively easily labeled with systemically delivered tomato lectin through the vessels by intravenous injection. In the case of other types of cells in dentin-pulp complex with no available endogenous genetic reporter, exogenous fluorescence labeling using additional fluorophore is required, which is not easily achievable in the intact tooth due to the hard tissue, enamel and dentin, limiting the direct injection of the fluorophore and low diffusion rate of a common exogenous fluorophore into the internal core of bulky tissue from the surface. To further expand the applicable topics in dental research using the optical clearing technique, these limitations need future development. Albeit these limitations, the optical clearing-based 3D cellular-level visualization can be a highly useful and efficient approach to comprehensively analyze the cellular behaviors of specific types of pulpal cells in normal and pathological conditions in a quantitative manner.

## 4. Materials and Methods

### 4.1. Animal Model

CD11c-YFP transgenic mice were kindly provided by Dr. Choi, Jae-Hoon at Hanyang University. CX3CR1-GFP transgenic mice (Stock no. 005582) and CSF1R-GFP transgenic mice (Stock no. 018549) were purchased from the Jackson Laboratory (Bar Harbor, ME, USA). Mice (total, *n* = 18; CD11c-YFP, *n* = 16; CX3CR1-GFP, *n* = 1; CSF1R-GFP, *n* = 1) used in this study were maintained in a specific pathogen-free facility of KAIST Laboratory Animal Resource Center. For experiments, 8–12 weeks old male mice (20~30 g) were used. Induction of acute pulpitis was performed with mice (*n* = 6) anesthetized by intraperitoneal injection of a mixture of Zoletil (30 mg/kg) and xylazine (10 mg/kg). Under the anesthesia, the mouse was placed on a stereotaxic plate to fix the maxillary incisors and mandibular incisors. The mesial cusp surface of the left-side maxillary 1st molar crown was gradually ground to expose dentin by using a dental drill bur (Strong 207A, Saeshin, Daegu, Korea). The irritated site was cooled by spraying PBS in a 31G syringe. After air-drying, the irritated site was etched with 37% phosphoric acid gel (Fine-Etch 37%, Spident, Incheon, Korea) for 5 min for widening the dentinal tubules. And the irritated site was washed with PBS and dried again [14,15,18]. All procedures were performed under dissecting microscope. Right-side maxillary 1st molar was utilized as a normal molar. Twelve h after the treatment, the irritated molars (*n* = 6) and normal molars (*n* = 6) were harvested and then optically cleared as described in 4.2. All animal experiments were performed in accordance with the standard guidelines for the care and use of laboratory animals and were approved by the Institutional Animal Care and Use Committee (IACUC) of KAIST (protocol no. KA2018-65). All procedures were performed under anesthesia, and all efforts were made to minimize the suffering of the animal. All animal experiments and reports of this work adhere to the ARRIVE Guidelines. 

### 4.2. Tooth Optical Clearing

Mice were anesthetized with an intraperitoneal injection of a mixture of Zoletil (30 mg/kg) and xylazine (10 mg/kg), and sacrificed with intracardiac perfusion with phosphate-buffered saline (PBS; LB004-02, Welgene, Daegu, Korea) and 4% wt/vol paraformaldehyde (PFA; BPP-9016, T&I, Gangwon, Korea, diluted in PBS). Dylight 649 conjugated tomato lectin (DL-1178, Vector Laboratories, Burlingame, CA, USA) were intravenously injected to fluorescently label blood vessels at 3 h before the perfusion. After the perfusion, teeth were harvested and washed by PBS for 1 min, and then further immersed in 4% PFA at 4 °C for 1 day. Then, teeth were immersed in 0.5 M ethylenediaminetetraacetic acid (EDTA; pH 8.0; CBE002, LPS solution, Daejeon, Korea) at 37 °C for 4 days with daily change of EDTA solution on a shaker [43]. Samples were washed with Dulbecco’s phosphate-buffered saline (DPBS; LB001-02, Welgene, Daegu, Korea) for at least more than 20 min, followed by 80% wt/vol ethanol (EtOH; CAS 64-17-5; 4022-4100, Daejung, Gyeonggi, Korea, diluted in distilled water; DW) for 1 day at room temperature. As an optical clearing agent (OCA), the BABB solution was made by mixing the vol/vol rate of one and two of Benzyl Alcohol (402834, Sigma, St. Louis, MO, USA) and Benzyl Benzoate (B6630-1L, Sigma, St. Louis, MO, USA). Dehydrated teeth by ethanol were immersed in an EP tube with peroxide-free BABB, which was made with 25% of Aluminum Oxide (Al_2_O_3_; 199443, Sigma, St. Louis, MO, USA) in BABB solution and taking out supernatant after centrifuging mixed solvents at 2000× *g* for 10 min [23]. Then the tube containing teeth is fixed on a rotator and kept at room temperature for 1 day.

### 4.3. Immunostaining

Immunostaining with DMP-1 was conducted as follows [44]. Extracted teeth samples were immersed in 4% PFA at 4 °C for 1 day. After washing with PBS, samples were incubated in 0.5M EDTA for 4 days at 37 °C with daily change of solution and then washed with DPBS for at least more than 20 min. Decalcified samples were bisected in the sagittal direction and incubated in DPBS, 0.3% Triton X-100, 20% DMSO, 0.3M glycine for 3 h at 37 °C. After washing using DPBS, 0.3% Triton X-100, samples were blocked using DPBS, 0.3% Triton X-100, 5% BSA, 10% DMSO (blocking solution) for overnight at 37 °C. The blocked samples were incubated with DMP1 antibody (1:200, sheep polyclonal, no. AF43861, R&D systems, Minneapolis, MN, USA) in blocking solution for 1 day at 37 °C. The immunolabeled samples were washed, and incubated with Alexa Fluor 647-conjugated anti-sheep IgG antibody (1:500, no. A-21448, Invitrogen, Carlsbad, CA, USA) in blocking solution for 2 days at 37 °C. After washing, samples were incubated with 4′,6-diamidino-2-phenylindole (DAPI) in DPBS to stain the nuclei for 3 h at RT. In all steps, samples were on a rotator or shaker. Samples were stored at 4 °C before confocal imaging. For immunostaining of tooth samples with Nestin, decalcified samples were embedded in paraffin. All embedded tissue samples were serially sectioned on the sagittal plane by 4 µm and mounted on silanized slides (no. 081001, Marienfeld, Lauda-Königshofen, Germany). The sections were treated with PBS, 0.5% Triton X-100 for permeabilization. After washing with DPBS, sections were blocked using DPBS and 2% BSA (blocking solution) for 30 min at RT. The blocked samples were incubated with Nestin antibody (1:100, mouse monoclonal, no. MA1-110, Invitrogen, Carlsbad, CA, USA) in DPBS, 2% BSA for 2 h. Subsequently, Cy3-conjugated anti-mouse IgG antibody (1:1000, no. AP124C, Millipore, Darmstadt, Germany) was added. The chromosomal DNA in the nucleus was stained with DAPI. Sections were stored at 4 °C before confocal imaging.

### 4.4. Imaging System and Analysis

To visualize the optically cleared tooth and the immunolabeled tooth at a 3-dimensional cellular level, a custom-built laser-scanning confocal microscope was used [22,45]. For fluorescence detection of 4′,6-diamidino-2-phenylindole (DAPI), YFP, and Dylight 649 conjugated tomato lectin or Alexa Fluor 649-conjugated antibody, three laser modules with wavelengths at 405 nm, 488 nm, and 640 nm and three high-sensitive photomultiplier tubes (PMT) equipped with band-pass filters (FF01-442/46, FF02-525/50, FF01-685/40, Semrock, Rochester, NY, USA) were used. To detect the dentinal collagen, a custom-built two-photon microscope was used [46]. For simultaneous visualization of second harmonic generation signal from dentinal collagen and YFP, a 140 fs pulsed Ti:sapphire laser tuned at 920 nm (Chameleon-Vision S, Coherent, Santa Clara, CA, USA) and two PMTs equipped with band-pass filters (FF01-445/20, FF01-525/45, Semrock, Rochester, NY, USA) were used. ImageJ (NIH, Bethesda, MD, USA) [47] was used to obtain a maximal intensity projection image from Z-stack image data. 3D-rendered image and animation movies were made with IMARIS (Bitplane, Zürich, Switzerland) [48]. Statistical analyses were conducted by using Prism software (GraphPad, San Diego, CA, USA) [49]. Statistical significance was identified by the two-tailed unpaired *t*-test or two-tailed paired *t*-test. 

## Data Availability

The data presented in this study are available on request from the corresponding authors.

## Supplementary Materials

The following are available online at https://www.mdpi.com/article/10.3390/ijms222312683/s1, Figure S1: Photograph for maxilla and mandible before and after optical clearing, Figure S2: CX3CR1+ and CSF1R+ cells extending cytoplasmic processes into dentinal tubules, Video S1: 3D reconstruction of optically cleared 1st maxillary molar of CD11c-YFP mouse showing CD11c+ cell extending long cytoplasmic process into the dentinal tubule, Video S2: 3D reconstruction of optically cleared 1st maxillary molar of CD11c-YFP mouse showing CD11c+ cell extending long cytoplasmic process into the dentinal tubule, Video S3: Z-stack images of irritated 1st maxillary molar of CD11c-YFP mouse. Video S4: 3D reconstruction of irritated 1st maxillary molar of CD11c-YFP mouse showing the spatial arrangement of pulpal CD11c+ cells in a barrier-like formation, Video S5: 3D reconstruction of irritated 1st maxillary molar of CD11c-YFP mouse.
