# Supplementary figures and images for: 3D Visualization of Dynamic Cellular Reaction of Pulpal CD11c+ Dendritic Cells against Pulpitis in Whole Murine Tooth
Source: Int J Mol Sci. 2021 Nov 24;22(23):12683. doi: 10.3390/ijms222312683 (PMC8657593; doi:10.3390/ijms222312683)

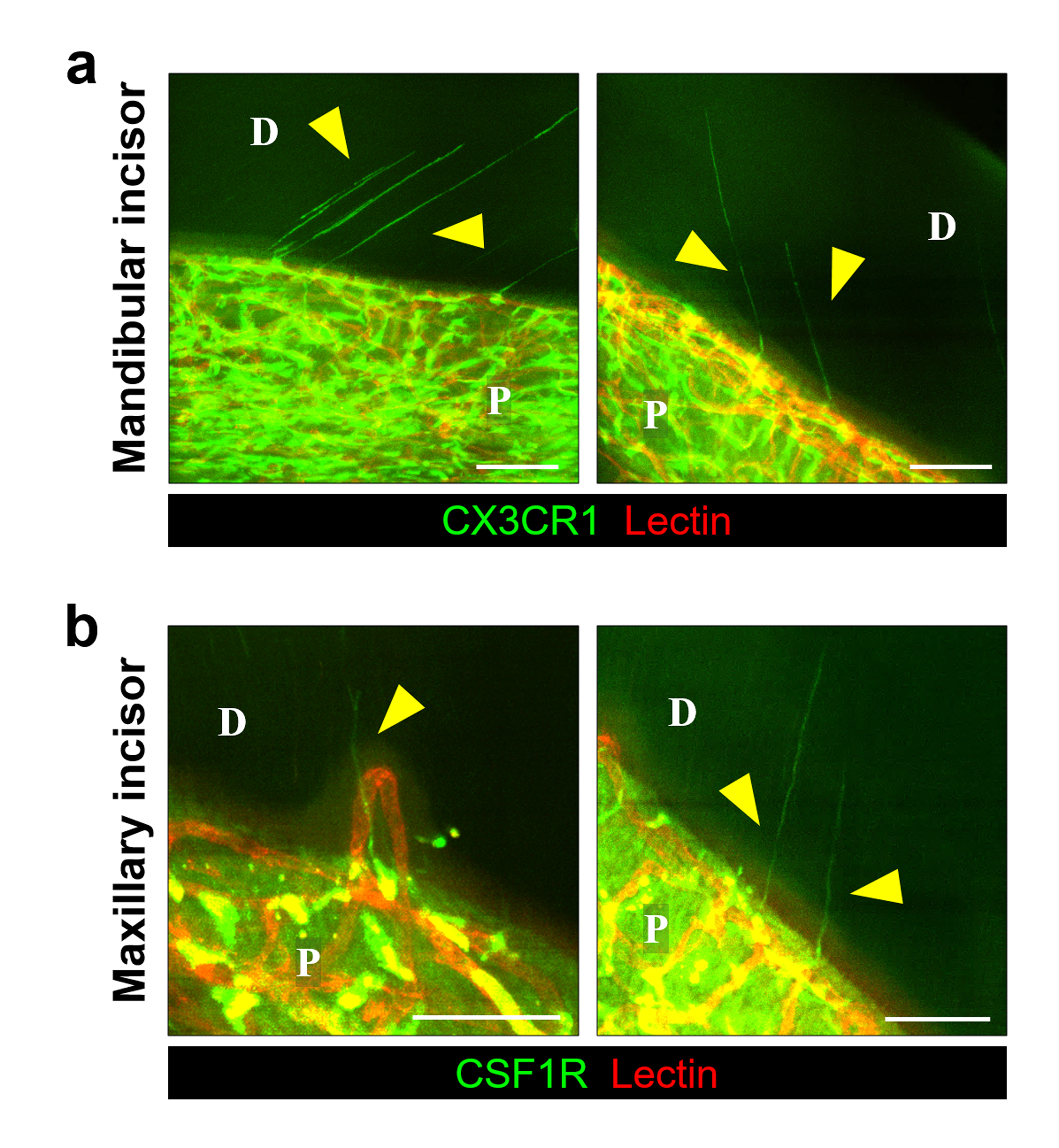

Supplement: Supplementary file 1 [file ijms-22-12683-s001.zip › Supplementary Figure S2.tif]

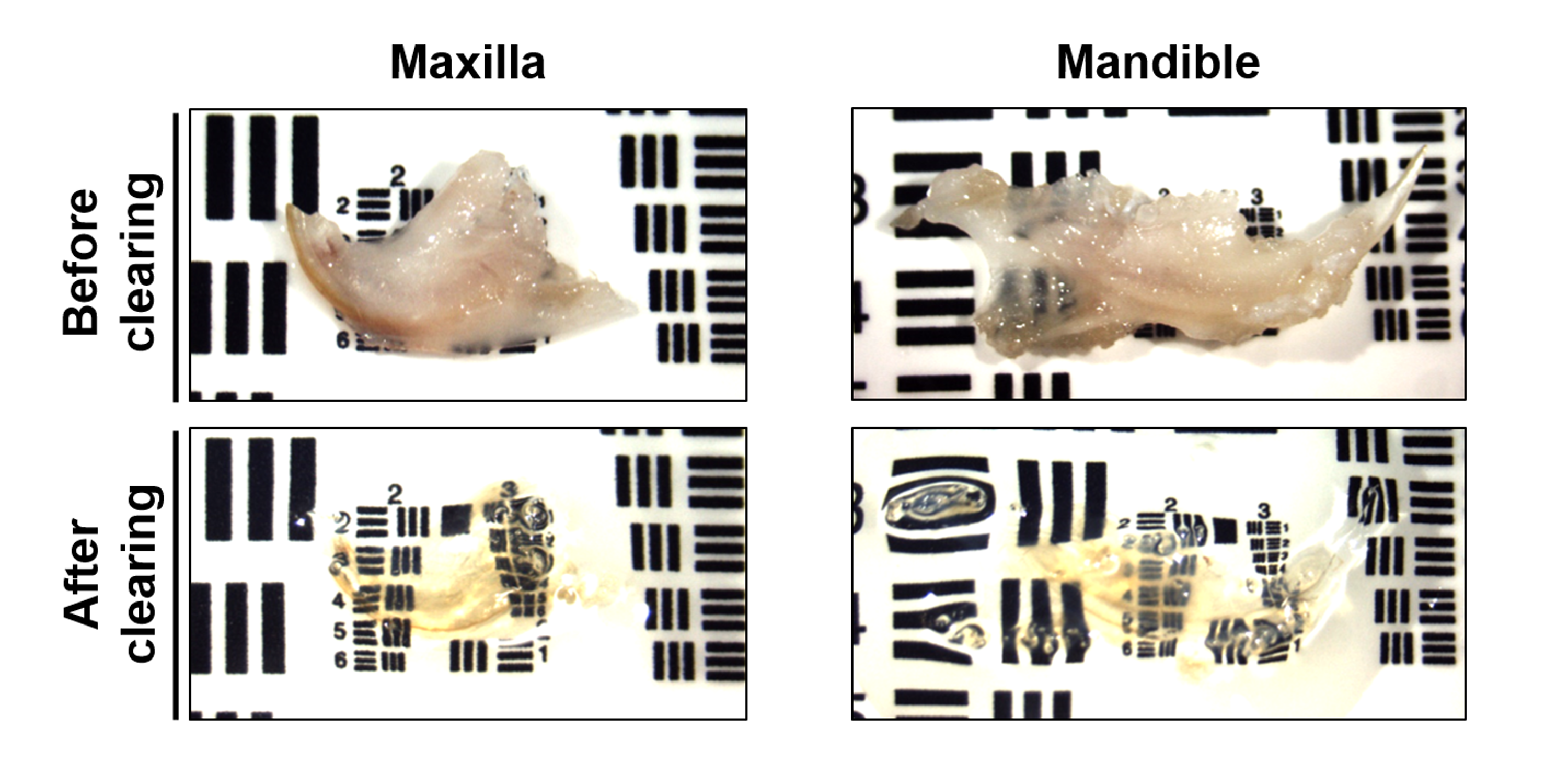

Supplement: Supplementary file 1 [file ijms-22-12683-s001.zip › Supplementary Figure S1.tif]
